# Mesoscale analysis of failure in quasi‐brittle materials: comparison between lattice model and acoustic emission data

**DOI:** 10.1002/nag.2363

**Published:** 2015-03-26

**Authors:** David Grégoire, Laura Verdon, Vincent Lefort, Peter Grassl, Jacqueline Saliba, Jean‐Pierre Regoin, Ahmed Loukili, Gilles Pijaudier‐Cabot

**Affiliations:** ^1^ Laboratoire des Fluides Complexes et leurs Réservoirs, LFC‐R UMR5150Université de Pau et des Pays de l'AdourAllée du Parc MontauryF64600AngletFrance; ^2^ University of GlasgowGlasgowU.K.; ^3^ GeM – Institut de Recherche en génie civil et mécaniqueEcole Centrale de NantesNantesFrance

**Keywords:** mesoscopic model, fracture process zone, damage, quasi‐brittle materials, experimental, acoustic emission

## Abstract

The purpose of this paper is to analyse the development and the evolution of the fracture process zone during fracture and damage in quasi‐brittle materials. A model taking into account the material details at the mesoscale is used to describe the failure process at the scale of the heterogeneities. This model is used to compute histograms of the relative distances between damaged points. These numerical results are compared with experimental data, where the damage evolution is monitored using acoustic emissions. Histograms of the relative distances between damage events in the numerical calculations and acoustic events in the experiments exhibit good agreement. It is shown that the mesoscale model provides relevant information from the point of view of both global responses and the local failure process. © 2015 The Authors. International Journal for Numerical and Analytical Methods in Geomechanics published by John Wiley & Sons Ltd.

## 1 Introduction

Fracture of quasi‐brittle materials such as concrete or rocks is characterized by a stress‐free macro‐crack surrounded by a damage zone. At the tip of the macro‐crack and ahead lies the so‐called fracture process zone (FPZ), which is a region of the material undergoing distributed damage. The size of the FPZ in these heterogeneous materials is large enough to influence the mechanical behaviour of the structure significantly. The understanding of the evolution of the FPZ size in such heterogeneous materials is still an open issue in the community. It is commonly accepted that this FPZ size does not depend on the structural size but is rather controlled by the local heterogeneities in the material as well as by the geometry of the specimen and the stress state. Therefore, size effect, understood here as the dependence of the dimensionless nominal strength of a structure on its size, is observed (e.g. when geometrically similar structures are compared; e.g. 1).

Experimentally, this damage zone may be characterized with the help of several direct and indirect techniques. Among the possible indirect techniques, energy‐based analyses involving redistribution of stresses in the FPZ have served as arguments for the justification of size effect (e.g. 2). Bažant and Pijaudier‐Cabot 3 determined the FPZ size experimentally as the ratio of the fracture energy to the energy dissipated per unit volume during the failure process. With the advent of displacement field measurement techniques, such as digital image correlation, access to local field quantities in the vicinity of the crack became possible in order to characterize the FPZ, opening the way for comparisons with results obtained from nonlinear models and calibration of constitutive models that describe the FPZ 4.

The localization of acoustic events that can be detected during crack propagation is another well‐established technique from which the FPZ can be visualized and characterized, in concrete 5, 6, 7, 8, 9 or rocks 10. The acoustic events generated during micro‐cracking are recorded and post‐processed in order to localize them with the help of time‐of‐flight algorithms. Hence, this technique provides information on the entire crack propagation process composed of distributed micro‐cracking and further coalescence into a macro‐crack. Haidar *et al.* 11 used a model mortar material to observe the correlation among the width of the FPZ measured by acoustic emission (AE) analysis, the parameters entering the description of size effect and the internal length used in nonlocal constitutive relations.

As far as modelling is concerned, continuum‐based approaches and discrete or mesoscale models are available. The first one involves a characteristic length that controls the size of the FPZ (e.g. the review by Bazant and Jirasek 12). In recent models 13, 14, 15, 16, an evolving, nonconstant, and nonlocal length internal length has been used, which was also assumed to be influenced by boundaries. Models with this advanced nonlocal length were capable of matching experimental results, which reported an influence of boundaries on the fracture energy 17. The second approach relies on a mesoscale description of the material and an explicit description of the heterogeneities in the material. Pioneering works, for example, by Van Mier and co‐workers, Herrmann and co‐workers or by Zubelewicz and Bazant 18, 19, 20, 21, have been extended to many problems, including dynamic fracturing in impact problems 22, cracking in coupled hydromechanical problems 23, modelling of phase interfaces at crack initiation 24, rock mechanics 25, concrete behaviour under high triaxial loading 26, fracture process of strain‐hardening cementitious composites 27, fracture of multiphase particulate materials 28, quasi‐static crack propagation 29, structural size effect 30, 31 and general failure behaviour of concrete 32, 33. The structural size effect on notched bending beams is properly described using this second approach 30.

Grassl and co‐workers 34 demonstrated that mesoscale modelling was very efficient at describing not only size effect on the peak load but also the entire load deflection response of bending beams. Four geometrically similar sizes and three different notch lengths were considered. The experimental data obtained by Gregoire *et al.* 1 could be quite accurately described, once the model parameters at the mesoscale level had been calibrated for one notch length. In addition, the authors used this model for studying the incremental distribution of the dissipated energy densities, and they were able to track the evolution of the FPZ in the structure, depending on the size of the beams and on the boundary conditions.

The purpose of this paper is to provide an additional insight on the pertinence of the mesoscale model introduced by Grassl and Jirasek 35. The first direction aims at a further understanding of the fracture and damage process. Instead of looking at snapshots of the incremental energy dissipation due to damage, we shall look at the distribution of the distances between points undergoing damage in order to investigate possible correlations involved in the kinetics of its propagation. The second direction is a comparison between damage events in the computational model and AE data. Assuming that each damage event is directly related to an acoustic event, we will compare these two processes in the course of failure on notched and un‐notched bending beams.

This paper is organized as follows. After having briefly recalled in Section 2 the lattice model used in this paper, we proceed in Section 3 to the analysis of the distributions of distance between damage events within a loading step and compare numerical results from notched and un‐notched bending beams. Section 4 presents the experimental apparatus used for the localization of acoustic events and the data obtained. The comparisons between results computed from the lattice model and AE data are presented in Section 5.

## 2 Lattice Model

The 2D plane‐stress lattice model proposed first by Grassl and Jirasek 35 is briefly presented in this section. The lattice is made from beam elements and idealizes the mesostructure of concrete as a set of three different components: *aggregates*, *matrix* and the *interface* between them. The following assumptions are used:

- Aggregates are described as circular inclusions. Aggregates with a diameter *φ* greater than a fixed diameter value *φ*_min_ are described explicitly. Their size distribution follows the grading of the concrete mixture, and their spatial location is given by a random distribution defined by the cumulative distribution function proposed in reference 35. Aggregates overlapping is avoided.
- Fine aggregates are not described. They are included in the matrix, which is an equivalent homogeneous material (made of cement paste and fine aggregates). Disorder due to the heterogeneity of the matrix that contains small aggregates is still kept, however, in the form of a correlated random distribution of mechanical properties. The correlation length is independent from the fineness of the lattice and therefore provides results independent of lattice element size independent 23.
- The large aggregate is elastic. The matrix material follows an isotropic – scalar – damage model.
- Each aggregate is surrounded by an interface of thickness equal to one lattice element length, which is endowed with a special constitutive relation. This interface is meant to represent the interfacial transition zone (ITZ) in concrete. Its constitutive model is similar to that of the matrix, with different constants because the ITZ is usually weaker than the matrix.

**Figure 1:**
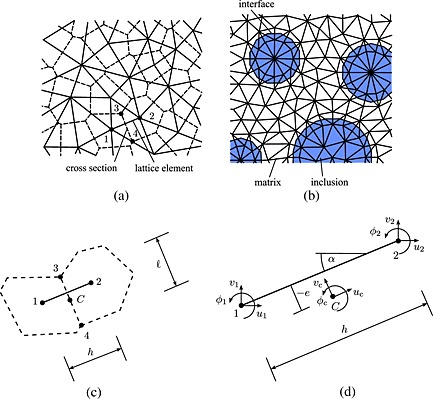
(a) Set of lattice elements (solid lines) with middle cross sections (dashed lines) obtained from the Voronoi tessellation of the domain. (b) Arrangement of lattice elements around aggregates. (c and d) Lattice element in the global coordinate system (reproduced from 34).

Once the largest aggregates have been placed randomly within the sample, the matrix is meshed by randomly locating nodes in the domain, such that a minimum distance *d*_min_ is enforced. The lattice elements result then from a Delaunay triangulation (solid lines in Figure 1(a)), whereby the middle cross sections of the lattice elements are the edges of the polygons of the dual Voronoi tessellation (dashed lines in Figure 1(a)). By contrast, the nodes located on both sides of an interface are not randomly distributed but placed at a special location in such a way that the edges of the Voronoi polygons define the interface between the aggregates and the mortar (Figure 1(b)).

Each node has three degrees of freedom: two translations (*u*,*v*) and one rotation (*φ*) as depicted in Figure 1(d). In the global coordinate system, the degrees of freedom of nodes 1 and 2, denoted as *u*_*e*_=(*u*_1_,*v*_1_,*φ*_1_,*u*_2_,*v*_2_,*φ*_2_)^*T*^, are linked to the displacement jumps in the local coordinate system of point *C*, *u*_*c*_=(*u*_*c*_,*v*_*c*_,*φ*_*c*_)^*T*^, by the following relation:

(1) $$u_{c}=Bu_{e}$$

where

(2) $$B=\left[\begin{array}{cccccc} -\cos\alpha & -\sin\alpha & -e & \cos\alpha & \sin\alpha & e\\ \sin\alpha & -\cos\alpha & -h/2 & \cos\alpha & \sin\alpha & -h/2\\ 0 & 0 & \sqrt{I/A} & 0 & 0 & -\sqrt{I/A} \end{array}\right]$$

where *A* is the element cross‐sectional area and *I* its second moment (refer to 34 for details).

Point *C* is located at the centre of the middle cross section of the element as represented in Figure 1(c) and (d). Matrix **B** depends on the orientation *α* of the element in the global coordinate system, the distance *e* between point *C* and the segment relating nodes 1 and 2, the distance *h* between two nodes, the element cross‐sectional area *A* and its second moment *I*. The strains *ϵ* = (*ϵ*_*n*_,*ϵ*_*s*_,*ϵ*_*φ*_)^*T*^ associated with the displacement *u*_*c*_ at point *C* are

(3) $$\epsilon =\frac{u_{c}}{h}={\left(\epsilon_{n},\epsilon_{s},\epsilon_{\varphi }\right)}^{T}$$

where *h* is the distance between the two nodes of one lattice element. The stresses *σ* = (*σ*_*n*_,*σ*_*s*_,*σ*_*φ*_)^*T*^ are related to the strains *ϵ* following the mechanical constitutive relation at the lattice level, here an isotropic damage model to be described further. The subscripts *n* and *s* refer to the normal and shear components of the strain and stress vector. The (secant) stiffness matrix **K** of the lattice element is defined as follows:

(4) $$K=\frac{A}{h}B^{T}DB$$

where **D** is the material stiffness matrix computed at point *C*.

The same isotropic damage model (Eq. (5)) is used to describe the mechanical response of the lattice element within the ITZ and the mechanical response of the matrix:

(5) $$\sigma ={\left(\sigma_{n},\sigma_{s},\sigma_{\varphi }\right)}^{T}=(1-\omega )D_{e}\epsilon =(1-\omega )\bar{\sigma }$$

where *ω* is the damage variable, **D**_*e*_ is the elastic stiffness and $\bar{\sigma }={\left({\bar{\sigma }}_{n},{\bar{\sigma }}_{s},{\bar{\sigma }}_{\varphi }\right)}^{T}$ is the effective stress. The elastic stiffness

(6) $$D_{e}=\left[\begin{array}{ccc} E & 0 & 0\\ 0 & \mathrm{\gamma E} & 0\\ 0 & 0 & E \end{array}\right]$$

depends on model parameters *E* and *γ*, which control Young's modulus and Poisson's ratio of the equivalent continuum 36. Equations (2) and (6) were chosen so that, for *h* = *ℓ* and *e* = 0, the stiffness matrix **K** reduces to the Bernoulli beam stiffness matrix 37.

The equivalent strain is then calculated from Eq. (7) where *ϵ*_0_, *c* and *q* are model parameters.

(7) $$\epsilon_{\mathrm{eq}}=\frac{1}{2}\epsilon_{0}(1-c)+\sqrt{{\left(\frac{1}{2}\epsilon_{0}(c-1)+\epsilon_{n}\right)}^{2}+\frac{c\gamma^{2}\epsilon_{s}^{2}}{q}}$$

The expression for the damage parameter *ω* is derived by considering pure tension, where the softening curve under monotonically increasing tensile strain is chosen to be of the exponential type:

(8) $$\sigma_{n}=f_{t}\exp\left(-\frac{w_{\mathrm{cn}}}{w_{f}}\right)$$

where *w*_*cn*_=*ω* *h* *ϵ*_*n*_ is the crack opening and *w*_*f*_ is the initial slope of the softening curve, which is related to the mesolevel fracture energy as *G*_*f*_=*f*_*t*_ *w*_*f*_. This stress–strain law can also be written, for pure traction, as a function of the damage variable as

(9) $$\sigma_{n}=(1-\omega )E\epsilon_{n}$$

In pure tension, the nominal stress is limited by the tensile strength (*f*_*t*_=*E* *ϵ*_0_), and thus, by using these two expressions of *σ*_*n*_, Eqs. (8) and (9), one obtains the expression, which governs the evolution of the damage variable *ω*:

(10) $$(1-\omega )\kappa =\epsilon_{0}\exp\left(-\frac{\mathrm{\omega h\kappa}}{w_{f}}\right)$$

where *ϵ*_*n*_ has been replaced by *κ*, which is a history‐dependent variable determined by Eq. (11a) with the Kuhn–Tucker loading–unloading conditions (11b):

(11a) $$f(\epsilon ,\kappa )=\epsilon_{\mathrm{eq}}(\epsilon )-\kappa$$

(11b) $$f\leq 0,\;\dot{\kappa }\geq 0,\;\dot{\kappa }f=0$$

The elastic constants and the model parameters in the damage models are calibrated from an inverse analysis technique. The material constants defining the mechanical behaviour of three material components are usually calibrated assuming certain ratios of their respective mechanical properties. The ITZ has a smaller tensile strength and a smaller fracture energy compared with the matrix. Typically, it has half the strength and half the fracture energy of mortar in the calculations. Experimental results for these ratios are reported in the literature (e.g. 18, 38).

## 3 Numerical Analysis of Fracture of Bending Beams

**Figure 2:**
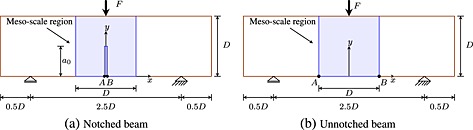
Geometries of a three‐point bending test for (a) notched and (b) un‐notched beams.

Figure 2(a) and (b) presents a schematic drawing of the notched and un‐notched beams considered in the present study. The geometry and applied loads correspond to the experiments reported in reference 1 and modelled numerically by Grassl and co‐workers 34. Four different sizes of geometrically similar specimens were considered, along with three notch lengths: *a* = 0 (un‐notched, so‐called UN), *a* = 0.2*D* (fifth‐notched, so‐called FN) and *a* = 0.5*D* (half‐notched, so‐called HN). For a detailed presentation of the experiments, refer to 1.

The analyses were controlled by the crack mouth opening displacement (CMOD), which is the relative horizontal displacement of the points A and B shown in Figure 2(a) and (b). For the notched specimens, the points were located at the end of the notch. For the un‐notched specimens, the two points were apart a distance equal to the beam depth *D*, because the location of the fracture process zone initiating from the surface was indeterminate.

Additional calculations on geometries that were not tested experimentally were performed in the present study in order to investigate beams of different sizes but with a different notch length, so that the ligament length is kept constant. This is motivated by the results obtained in 34, which show that the distribution of energy dissipation during several load increments in the post‐peak regime seems to be independent of the notch type for a given ligament length. In order to have a ligament length of 200mm, we considered a beam of type UN having a depth of *D* = 200 mm and a beam of type FN with *D* = 250 mm. These beams are denoted as UN200 and FN250, respectively. The beams HN200, FN125 and UN100 correspond to a ligament length of 100mm. Beams HN100, FN62.5 and UN50 have a ligament length of 50mm.

Same as in the experiments, the out‐of‐plane thickness was kept constant for all sizes and all geometries at *b* = 50 mm. The notch thickness was fixed equal to zero for consistence with the experimental procedure, where the notch was moulded using a thin metal plate of constant thickness. The load and support reactions were applied by means of 5‐mm‐wide metallic plates.

In order to limit the computation time, the nonlinear mesoscale model is used in the middle part of each beam centred at mid‐span, where damage is expected, as shown in Figure 2(a) and (b). The remaining part of the beam is discretized with elastic lattice elements. In this region, the aggregates are not described explicitly. The mechanical response of this part of the lattice corresponds to that of the equivalent homogeneous material. The aggregate volume fraction corresponds to the experimental data, ¶ with a cut‐off for small sizes: *φ*_min_=5 mm. Fine aggregates are not explicitly described. They are included in the matrix, which is an equivalent homogeneous material made of cement paste and fine aggregates. Disorder due to the heterogeneity of the matrix is considered in the form of a correlated random distribution of mechanical properties. The correlation length is equal to 1mm. Details may be found in 34. The model parameters for the three components are summarized in Table 1. Young's moduli and the tensile strength of the matrix and the ITZ are obtained from the computation of the response of a tensile test. The other model parameters, *q*,*c* and *G*_*f*_, are taken from 34. They were obtained from the calibration of the load displacement response with experiments for a medium‐size bending (notched HN) beam. For each geometry, calculations were repeated with 10 different random fields of aggregates and mechanical properties.

**Table 1:** Model parameters.

	*E* (GPa)	*ν*	*f*_*t*_ (MPa)	*q*	*c*	*G*_*f*_ (N/m)
Matrix	44	0.33	3.8	2	10	86
Interface	58.7	0.33	1.9	2	10	43
Aggregate	88	0.33	—	—	—	—
Mean	63	0.33	—	—	—	—

Because we are basically repeating the calculations reported in reference 34, we shall discuss neither the global responses of the lattices nor the energy dissipation maps in the vicinity of the crack tip. Instead, we shall try to concentrate here on the kinetics of the failure process. For this, we are going to compute the relative distance between lattice elements undergoing damage during a load step. Later on, this will be referred to as *the distance between damage events*. The histogram of these relative distances should exhibit correlations because of the distribution of strain (and therefore the loading conditions) within the lattice and also correlations induced by interactions during failure. Such correlations have been investigated by Delaplace and co‐workers 39, 40 in the context of tensile tests on lattices, where similar considerations were made and for mode I fracture tests on the basis of the statistical distribution of avalanches of local failure events.

**Figure 3:**
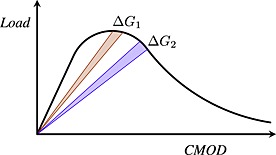
Sketch of the different histogram‐computing intervals on a typical force versus crack mouth opening displacement (CMOD) curve.

Histograms are computed from the distribution of the relative distances between points undergoing damage during an interval of dissipated energy Δ*G*_*f*_=12.5 × 10^−3^ J. This amount of dissipated energy is chosen according to the ones chosen in reference 34 to build energy dissipation maps. We consider one increment of dissipated energy Δ*G*_*f*_ between two steps of loading as depicted in Figure 3. The first increment, Δ*G*_1_, starts at the peak load, and the second one, Δ*G*_2_, starts 10 loading steps after Δ*G*_1_, in the softening regime (the tests are CMOD controlled with a CMOD step equal to Δ_CMOD_=2 × 10^−6^ m). The principle of post‐processing is simple: we track, during the increment Δ*G*_*f*_, all the *C* points (Figure 1(d)) corresponding to the lattice elements, where damage increases, and compute the distances between these *C* points. Then, we take the projections of the distances onto a horizontal axis (perpendicular to the crack propagation) and a vertical axis (in the direction of the crack propagation). Afterwards, the distribution of these distances is plotted. For comparison purposes, the distributions are normalized with respect to the total number of distances computed, and the distances are divided by the ligament length of each beam.

**Figure 4:**
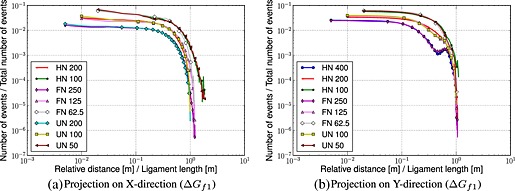
Histograms representing the evolution of the number of events versus the relative distances normalized by the ligament length – increment starting at peak (Δ*G*_*f*1_). FN, fifth‐notched; HN, half‐notched; UN, un‐notched.

**Figure 5:**
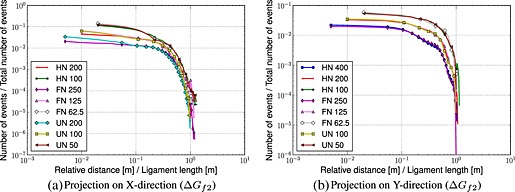
Histograms representing the evolution of the number of events versus the relative distances normalized by the ligament length – increment in the post‐peak regime (Δ*G*_*f*2_). FN, fifth‐notched; HN, half‐notched; UN, un‐notched.

Figures 4 and 5 show the results. Two comments can be made:

1. In the case of pure tension, one would expect that the histograms are composed of two parts 39: a relatively horizontal part corresponding to a random distribution of damage events above a correlation length and below a power‐law‐type distribution. This is not the case in a bending test. For small distances, a power law distribution should provide a linear regime in a log‐log plot. This is not what is observed. Instead, a rather smooth nonlinear curve is obtained. For large distances, the horizontal (constant) distribution cannot be observed because the strain gradient over the beam depth prevents the random distribution of events to develop (damage cannot occur in compression). The zone in which damage occurs within a loading increment is constrained by the strain gradient because of bending.
2. Although these histograms cannot be interpreted as easily as in a pure tension test, some striking results are obtained: when the ligament length is kept constant, the histograms fall onto the same curve. It means that they are the same for a beam of depth 200mm, with a notch length of 100mm, a beam of depth 125mm with a notch length of 25mm and an un‐notched beam of depth 100mm. The same is observed for other sizes, as long as the ligament length is kept constant. Once the localized failure is initiated, the failure process seems to be solely influenced by the ligament length. This feature was also observed by Grassl *et al.* 34, where dissipation maps were plotted, and a good agreement for beams with the same ligament length but different notch types was obtained.

To summarize, on the one hand, the analysis of the distribution of distances between damage events provides results that are consistent with previous lattice analyses carried out on the same geometries. On the other hand, the distributions themselves exhibit effects because of strain gradient and correlation of failure events that seem difficult to separate, as opposed to the case of pure tension. A simple interpretation of these histograms, in terms of extraction of a correlation length for instance, is not straightforward. Because a major objective of this work consists of determining the capacity of the mesomodel to catch up relevant information on the mechanical behaviour of the structure at the local and global scales, the previously mentioned histograms and the distribution of dissipated energy during fracture shall be compared with experimental data.

## 4 Experimental Study

Three‐point bending tests were performed on geometrically similar notched and un‐notched specimens made of the same concrete material. The experimental results presented hereafter are obtained from a campaign similar to the one presented by Gregoire *et al.* 1, which includes the localization of acoustic events during fracture additionally.

### 4.1 Material, specimen and test rig descriptions

**Figure 6:**
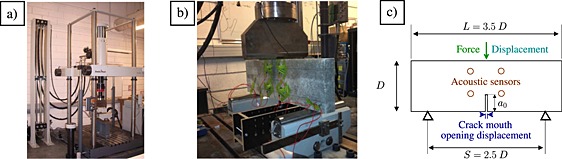
Photography of the servo‐hydraulic testing machine (a), zoom on the test rig (b) and sketch of the specimen geometry and measurable quantities (c).

The concrete formulation used here is based on a ready‐mix concrete mixture obtained from Unibeton (http://www.unibeton.fr) and detailed in Table 2. Detailed gradings of the sand, the aggregates and the mix are given in 1. After demoulding, the specimens were stored under water at 20°C. The characterization of their mechanical properties was made by compression and splitting (Brazilian) tests according to European standards (EN 12390‐1‐3‐6). Table 3 summarizes these mechanical properties. Because the concrete used for the present study is the same as the one used in 1, detailed information about the mechanical response of the material is not repeated. The testing rig used for the bending tests was a three‐point bending set‐up on a servo‐hydraulic testing machine (HB250, Zwick/Roell, Ulm, Germany; Figure 6(a) and (b)).

**Table 2:** Concrete mixture formulation.

Product	Designation	Mass (kg)
Sand	Cemex 0/4	40
Aggregates	Durruty 4/10	1140
Cement	Calcia CEM II/A	286
Admixture	Axim Cimplast 115	1
Water	Clarified water	179
Total		2346

**Table 3:** Concrete mean mechanical properties.

Compressive strength			Young modulus			Poisson ratio			Tensile strength		
*μ*	*σ*	*c*_*v*_	*μ*	*σ*	*c*_*v*_	*μ*	*σ*	*c*_*v*_	*μ*	*σ*	*c*_*v*_
(MPa)	(MPa)	(%)	(GPa)	(GPa)	(%)	(—)	(—)	(%)	(MPa)	(MPa)	(%)
42.3	2.8	6.6	37.0	0.9	2.4	0.21	0.02	8.7	3.9	0.2	6.0

*μ*, mean value; *σ*, standard deviation; *c*_*v*_=*μ*/*σ*, coefficient of variation.

**Figure 7:**
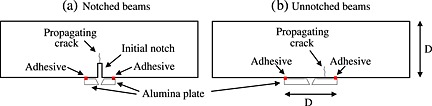
Crack mouth opening displacement measurement for (a) notched and (b) un‐notched beams (reproduced from 1).

Figure 6(c) presents a sketch of the specimen geometry and the different measurable quantities. Three HN200 half‐notched (*D* = 200 mm; *a*_0_=0.5*D*), three FN200 fifth‐notched (*D* = 200 mm; *a*_0_=0.2*D*), two UN200 un‐notched (*D* = 200 mm; *a*_0_=0 mm) and three UN100 un‐notched specimens (*D* = 100 mm; *a*_0_=0 mm) have been tested. The thickness was kept constant (50mm). All tests were CMOD controlled at an imposed velocity (*v*_CMOD_). Table 4 summarizes the different specimen dimensions and the experimental conditions. The CMOD measurement was achieved by recording the distance between two aluminium plates glued on the bottom of the surface beam separated by the initial moulded notch. On the un‐notched beams, these metallic plates are glued at a distance equal to a half depth from the middle of the beam to ensure that the crack initiates between the two plates. In this case, the measure is not a CMOD. The numerical simulations were performed accordingly. Figure 7 gives a representation of the notched and un‐notched beams and the corresponding positions of the aluminium plates. The CMOD was gradually increased until the complete failure of the structure.

**Table 4:** Specimen dimensions and experimental conditions.

Label	*D* (mm)	*a*_0_ (mm)	*v*_CMOD_ (μm/s)
HN200	200	100	0.3
FN200	200	40	0.3
UN200	200	0	0.3
UN100	100	0	0.2

FN, fifth‐notched; HN, half‐notched; UN, un‐notched.

### 4.2 Acoustic emission measurements

**Figure 8:**
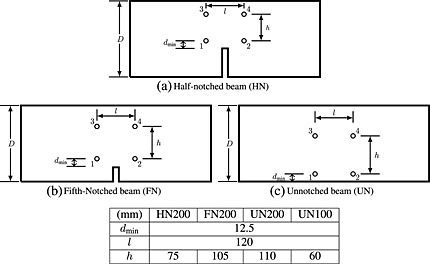
Position of the acoustic sensors for the three different beam geometries.

During the tests, acoustic events were recorded and localized. The AE system used in this study comprised an eighth‐channel MISTRAS system, a general‐purpose interface bus (PCI‐DISP4) and a PC for data storage analysis. Four acoustic transducers (resonant frequency of 150kHz) were placed around the expected location of the crack, on one side of the specimen. The AE event localization program relies on time‐of‐flight analysis and triangulation. The criterion used is that waves generated must reach at least three sensors. Then, the source location is determined by a 2D triangulation algorithm, which relies on AE arrival time and wave velocity. The details about AE setting parameters are given in 9. Transducers were installed so that a minimum distance of *d*_min_=1.25 cm to the location where the crack could appear was respected in order to minimize errors, which may occur when events are located near one sensor. Figure 8 shows the arrangement of the transducers for all the tested geometries.

The detected signals were then amplified with a 40‐dB gain differential amplifier in a frequency band from 20 to 120kHz. In order to limit the background noise, the signal detection threshold was set at a value of 35dB. The coupling between the transducer and the specimen is important in order to achieve a good accuracy of the localization of events. A thin layer of silicone grease was used to guarantee the correct transmission of acoustic signals between the beam and the transducer. The validation of both this coupling and the accuracy of the acoustic event localization followed the European standard NF EN 1330. It consists of verifying if the position of an on‐surface signal generated by the break of a short piece of pencil lead is correctly determined by the triangulation software. Thus, events were generated at several locations on the surface of each specimen, and the results from the localization software were compared with the true location of each event. A correct coupling is achieved when the accuracy of localization of these events is in the order of 4 mm.

## 5 Experimental Results and Comparison with Computations

### 5.1 Mechanical responses and acoustic emission

**Figure 9:**
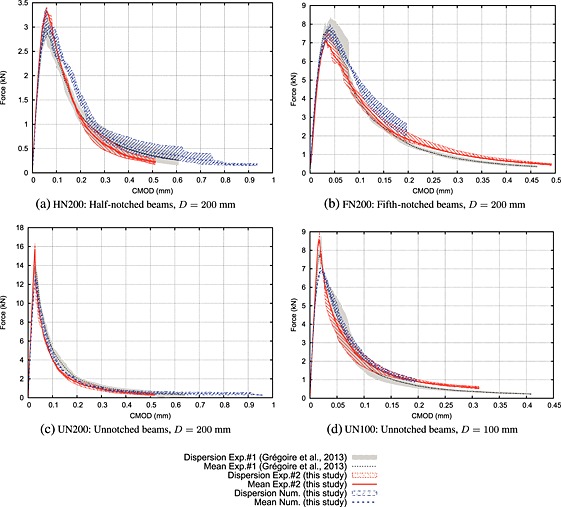
Force versus crack mouth opening displacement (CMOD) raw curves.

The experimental and numerical results in terms of force versus CMOD data are presented in Figure 9. Here, we compare the numerical results obtained with the mesomodel, denoted as ‘Num.’, the results reported by Grégoire *et al.* 1, denoted as ‘Exp.#1’, and the experimental results from our experimental work, denoted as ‘Exp.#2’. As expected, we observe that the experimental data points are in good agreement with the results obtained via the mesoscopic approach. This result is similar to the one obtained in 34, and it confirms that the experiments carried out with the AE analysis are indeed similar to the previous experiments reported in 1 and shows the repeatability of the experimental tests. Note that neither some fitting nor adjustments of the model parameters in the computation have been performed. The experiments have been performed using the same concrete formulation as in 1. The numerical simulations have been performed using the parameters presented in 34.

**Figure 10:**
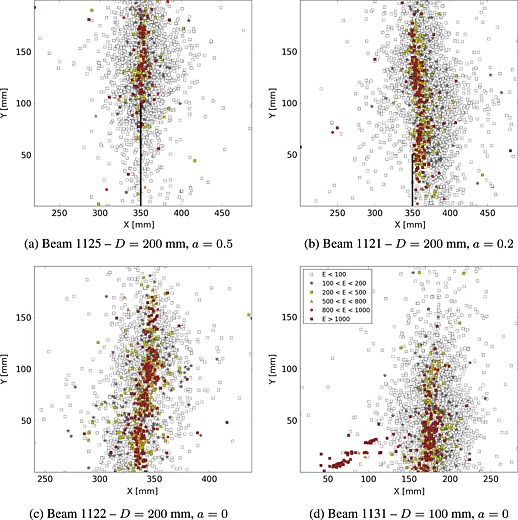
Distribution of acoustic events in different geometries of the beam – map of the distribution of energies.

Figure 10 shows the results of the cumulative locations of the acoustic events. The plotted points indicate the detected AE sources over the observation window centred at the notch. Events carry different energies, and we have plotted here all the events. The warmer/darker the marker of one event, the higher the acoustic energy (in black and white). One can filter the events and retain only those with a sufficiently large acoustic energy. These events should correspond to the macro‐crack propagating in the specimen. Such an analysis points out that the process begins with low‐energy events distributed in a diffuse way, followed by a concentration of events with an increased rate of dissipated energy (also 5). For notched specimens, the inception and the path of the macro‐crack is characterized by a strong concentration and by alignment of the most energetic events. For un‐notched specimens, acoustic events are spread on the bottom of the beam prior to the propagation of a single macro‐crack on which the most energetic events are recorded. The location of crack initiation results from a competitive effect between the strain gradient and the local distribution of weak defects.

### 5.2 Comparison of dissipation maps

#### 5.2.1 Methodology

Assuming that the acoustic energy recorded for each event is proportional to the energy dissipated during the corresponding damage event, it is possible to compare the dissipation maps during an increment of the load:

- On one hand, the dissipated energy during damage is obtained numerically from the mesoscale analysis. Maps of dissipated energy have been already computed in 34, 35, and we follow the same procedure. The domain to be analysed is first discretized with a square grid with a cell size of *d* = 2.46 mm. Within each cell, the energy dissipation due to damage is computed for each lattice element located in the cell. The dissipated energy in a single lattice element is calculated as $\Delta D_{d}=\mathrm{\Delta \omega Ah}\frac{1}{2}\mathrm{\epsilon D\epsilon}$. Here, Δ*ω* is the increment of damage parameter; *h*, *A*, *ϵ* and *D* were defined in Section 2. Then, we sum this energy dissipation for all lattice elements contained in the cell. When a lattice element crosses several cells, the energy is allocated in proportion to the element length within each cell. As discussed in reference 34, the extent of spatial distribution of energy density obtained from this method represents the fracture process zone.
- On the other hand, the maps of the distribution of acoustic energy within the same loading increments are computed according to the same discretization. Within an increment, the energy of all the event contained in the same cell is summed up. Because of the localization resolution by the AE technique, less acoustic events than numerical events are detected. Therefore, the size and the discretization of the load increments are determined to ensure that enough events are captured experimentally in order to achieve statistically representative experimental histograms.

**Figure 11:**
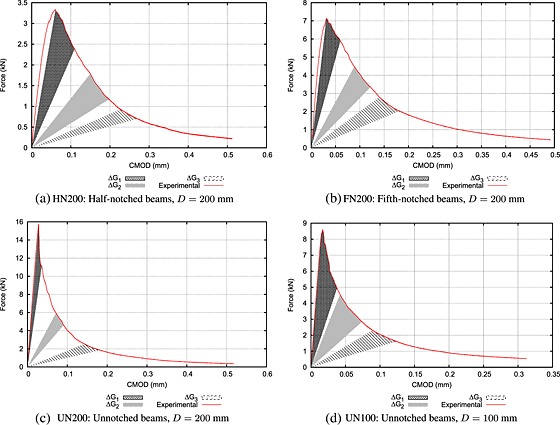
Different intervals of dissipated energy represented on the averaged force versus crack mouth opening displacement (CMOD) experimental curves.

In both approaches, the energy maps are averages from the same number of tests. Three *D* = 200 mm half‐notched, three *D* = 200 mm fifth‐notched, two *D* = 200 mm un‐notched and three *D* = 100 mm un‐notched specimens have been tested both experimentally and numerically. Three different loading increments are considered here corresponding to the same interval of dissipated energy (Δ_*G*1_=Δ_*G*2_=Δ_*G*3_=120 × 10^−3^ J). Figure 11 presents the different intervals sketched on the averaged force versus CMOD experimental curves. Note that the interval of dissipated energy is approximately 10 times larger than the one used in Section 3, when only numerical results were post‐processed. Indeed, this interval of dissipated energy is increased in order to achieve statistically representative histograms from experimental AE data.

#### 5.2.2 Influence of the size of the cell on the dissipation maps

Because the total energy dissipated within a cell is taken into account, the size of the cells has almost no influence on the dissipation maps. The size of the cells corresponds to the size of the discretization of the energy functional as in the numerical analysis of continuum functionals. However, a minimum size of discretization has to be respected because the energy functional is not continuous. Numerically, the minimum size corresponds to the lattice discretization. Experimentally, the minimum size corresponds to the resolution of the AE detection of the the acoustic sensors. Except for these minimums, the dissipation maps are similar whatever the size of the cells in the sense of the numerical analysis. The numerical minimum is far lower than the experimental minimum. Because the first goal of the paper is to test the relevance of the mesomodel by comparing the numerical results with experimental ones, we adopt the same discretization, which is driven by the experimental minimum. Numerical dissipation maps with a much lower cell size are presented alone in 34.

#### 5.2.3 Influence of the length of the interval of dissipated energy on the dissipation maps

The length of the interval of dissipated energies has a significative influence on the results. By taking into account the interval of dissipated energies, the results represent an average. Because our first goal is to characterize the growth and evolution of the fracture process zone, we aim at considering the shortest interval of dissipated energies to perform the analysis. However, and unfortunately, there are intrinsic minimums of internal lengths that have to be respected. The first minimum corresponds to the loading step. No information is acquired experimentally nor estimated numerically between two loading steps. The second minimum corresponds to the fact that a minimum of damage events has to be captured to perform the post‐processing. Numerically, there is almost no limitation because many damage events are acquired within a loading step. Experimentally, this is much more restrictive because only few acoustic events are acquired by AE technique. Therefore, the size of the interval of dissipated energies are determined to ensure that enough events are captured experimentally in order to achieve a statistically representative post‐processing. Because the first goal of the paper is to test the relevance of the mesomodel by comparing the numerical results with experimental ones, we adopt the same interval length, which is driven by the experimental minimum. Numerical dissipation maps with a lower interval of dissipated energy are presented alone in Section 3.

#### 5.2.4 Results

**Figure 12:**
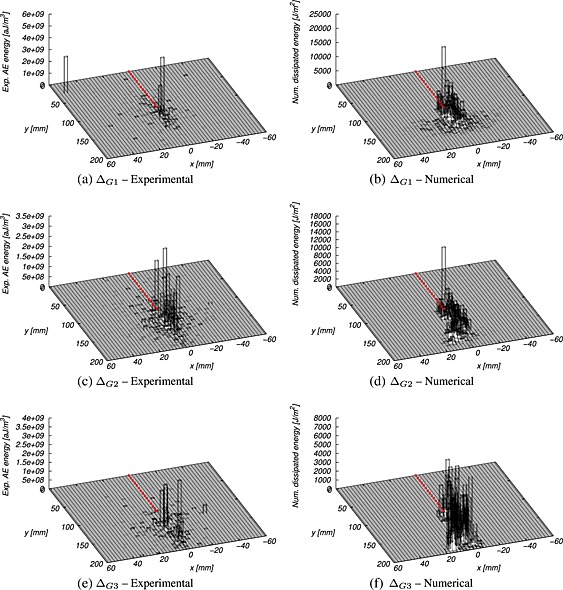
Energy maps during the different energy increments obtained from acoustic emission (AE) analysis (experimental) and mesoscale computations (numerical) for HN200 bending beam (long notch, size *D* = 200 mm). The prenotch is highlighted with a dashed line.

Figures 12 and B.1, B.2, B.3 in Appendix B present a comparison of average distributions of energy densities between numerical and experimental results for the three dissipated energy intervals considered and all geometries. We did not superimpose these 3D maps for the sake of clarity. Moreover, the energies involved are quite different. The acoustic energy is only a small part of the dissipated energy upon local fracture. The largest part is converted into the creation of free surfaces (cracks). Although the dissipated energy obtained numerically and the AE energy obtained experimentally are not the same, they are strongly related (e.g. 41), and a qualitative direct comparison makes sense. Note that the AE energy is expressed in attojoules by cubic metres, whereas the numerical dissipated energy is expressed in joules by square metres. Because the numerical simulations are 2D, the dissipated energy stands here for a unit metre in width.

**Figure B.2:**
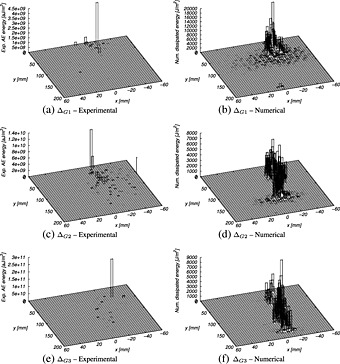
Energy maps during the different energy increments obtained from acoustic emission (AE) analysis (experimental) and mesoscale computations (numerical) for UN200 bending beam (no notch, size *D* = 200 mm).

At first, one can remark that experimental results exhibit a more pronounced scattering than numerical ones. AE acquisitions are filtered to avoid spurious detection. A low filter threshold (35dB) is preferred to ensure that all events associated with micro‐cracking are captured. At the same time, with a low value, disperse and low‐energy events may be taken into account, while they are not associated with micro‐cracking. This may be a source of experimental scattering. Despite this scattering, the extents of the energy maps (which represent the FPZ) are similar. At initiation (Δ_*G*1_), in notched beams (Figures 12 and B.1), most of the acoustic and fracture energies are dissipated in a localized region following the axis of the prenotch reaching the maximum value at the prenotch tip to become wider far from the notch, while energy decreases. In un‐notched beams (Figures B.2 and B.3), a similar trend is observed. The expected distributed damage prior to the onset of localized cracking is not observed, because the number of acoustic events is not large enough in order to obtain a representative distribution prior to the initiation of the macro‐crack. After initiation and during the crack propagation (Δ_*G*2_, Δ_*G*3_), similar trends are observed in notched and un‐notched beams.

**Figure 13:**
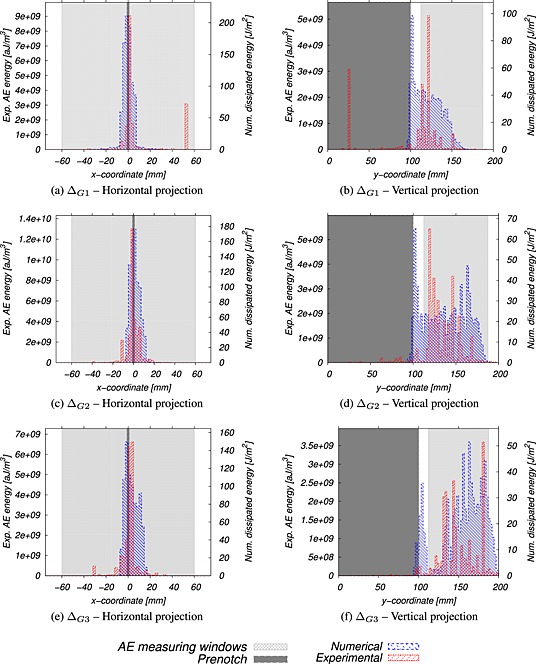
Horizontal and vertical projections of energy dissipation for HN200 bending beams (long notch, size *D* = 200 mm).

The similarity of the damage and AE maps is better observed by looking at projections of the energy maps in the horizontal and vertical directions. Figures 13 and C.1, C.2, C.3 in Appendix C showprofiles obtained for all beam geometries and all dissipated energy increments. Qualitatively, the profiles have the same extent. They are rather similar, although the experimental ones are more discrete, with successive peaks that can be related to the accuracy of the measurements and quantity of available data (the size of the aggregate may also have an influence). Note that the AE measuring windows corresponding to the area between the AE sensors are presented in all figures. Experimental events occurring outside the AE windows may not be localized accurately. Finally, the agreement between numerical and experimental data is rather correct for all beam geometries and all dissipated energy increments.

This similarity between the mesoscale results and the AE test data indicates that the computational model is not only capable of matching global experimental data (such as force versus CMOD curves) but also local data (such as the distribution of energy dissipation). Comparisons between the distribution of relative distances between events will further document this conclusion. Numerically, a damage event corresponds to a lattice material point (point C in Figure 1) undergoing damage during a load step. Experimentally, a damage event corresponds to a material point producing AEs upon failure, which have been detected (and then localized by triangularization) by at least three acoustic sensors. The distance between these events occurring during the considered loading interval defines the distance between damage events.

### 5.3 Comparison of histograms of relative distances between damage events

**Figure 14:**
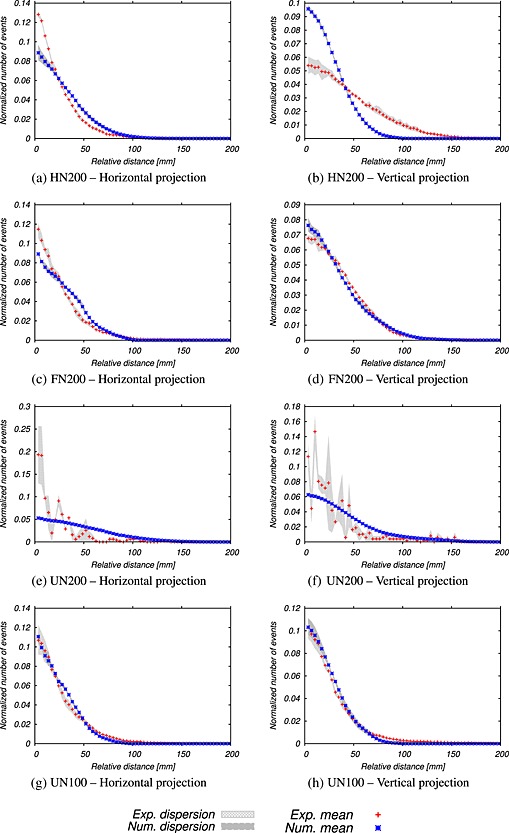
Horizontal and vertical projections of the experimental and numerical histograms at peak (first energy increment Δ_*G*1_ in Figure 11).

Figures 14, D.1 and D.2 in Appendix D present the comparisons of histograms of relative distances between damage events at peak and during the softening phase, respectively (see the increments Δ_*G*1_, Δ_*G*2_ and Δ_*G*3_ in Figure 11). Globally, the agreements are rather good, also given the fact that no adjustable parameter is included in the numerical results.

**Figure D.1:**
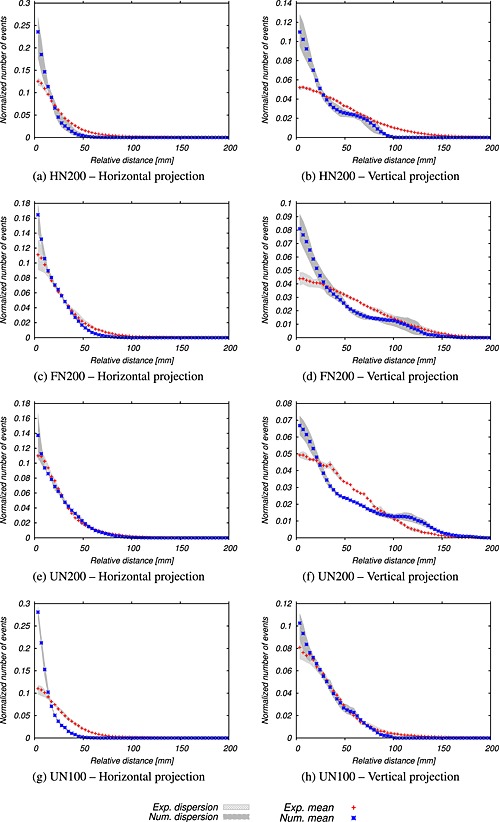
Horizontal and vertical projections of the experimental and numerical histograms in the softening phase (second energy increment Δ_*G*2_ in Figure 11).

Particularly, we may notice that the agreement is really good at a peak where the numerical and experimental histograms are quite similar, whatever the geometry (Figure 14). A large scattering is observed during the experimental UN200 test. Indeed, only few damage events have been detected with the AE apparatus at the peak for this geometry on both samples. Therefore, it enlarges the scattering, especially on small relative distances, which are less detected during the experiments. During the softening phase (Figures D.1 and D.2 in Appendix D), the agreements are still acceptable, but differences between numerical and experimental histograms may be observed. Small relative distances may not be detected with the AE apparatus during the experiments, whereas all damage events are recorded numerically. Therefore, the weight of the small relative distances is more important in the numerical histograms than in the experimental ones. This is particularly the case in the horizontal direction and when the crack propagates far away from the notch tip (stage 3).

Finally, the numerical and experimental histograms are quite similar, which proves that the mesoscale approach is capable of capturing the local aspect of the fracturing process.

## 6 Conclusion

We have presented a detailed analysis of the cracking process in three‐point bending specimens from both experimental and numerical points of view. The computational model is a mesoscale, lattice‐based approach, which already proved to be able to capture size effect test data as well as force versus CMOD response for notched and un‐notched bending beams. Experiments coupled with AE analyses provided global responses of the same bending beams and local data in the form of the distribution of the acoustic events and its evolution in the course of fracture.

The following concluding statements can be made:

- The analysis of the distribution of the relative distances between damage events in the computational model shows that the histograms depend on the ligament length. For the same ligament length, whatever the notch length in the beam, the histograms are the same. Grassl and co‐workers 34 observed the same trend by looking at maps of energy dissipated.
- Contrary to the case of direct tension, these histograms cannot be interpreted easily because the effect of the strain gradient in bending beams cannot be easily separated from the interaction between damage events that may develop in the course of fracture. Hence, the paper has been more dedicated to comparison between experimental and numerical data on the same set of geometries and loading conditions.
- The energy dissipated due to damage in the computational model and the acoustic energy recorded during the experiments provide maps that are qualitatively very similar.
- The histograms of the distances between damage events in the computational model and between acoustic events in the experiments agree quite closely. These histograms are computed for events located within a loading increment after the peak load. Similar results in the softening regime have been presented.
- At this stage, a restrictive 2D mechanical mesomodel has been used to analyse inherent 3D AE data. We are currently developing a 3D version of this mesomodel. We will then be able to analyse the 3D effects on the numerical results. However, we do not expect drastic differences because the thickness of the beam is small (50mm) and the AE analysis is itself 2D. The failure process is of course 3D, but four acoustic sensors have been placed on only one side of the beams, and therefore, the analysis of the AE localization is only 2D. A whole combined 3D study (3D mesomodel and 3D AE localization) should be performed to analyse the 3D effects, especially the shape of the FPZ on the surface boundaries.

Discrepancies between experimental and numerical results may be due to the following:

- the experimental inaccuracy in the acoustic event localization;
- the AE acquisition filtering;
- a 2D numerical analysis and a 2D AE analysis of a 3D failure process;
- the relatively few number of specimens tested experimentally; and
- the relatively few number of damage events acquired experimentally and, consequently, the relatively large intervals of dissipated energy considered to achieve a statistically representative post‐processing.

However, and despite all these sources of discrepancies, the overall agreement that has been observed between the mesoscale approach and the experimental data demonstrates that the mesoscale approach is capable not only of providing consistent global responses (e.g. force versus CMOD responses) but also capturing the local failure process realistically. The agreement between the distributions of the relative distances between damage events shows that the mesoscale model depicts the FPZ and its evolution during failure in a very consistent way compared with AE data. This conclusion opens the path for further analyses of the fracture process, solely based on numerical analyses with the mesoscale model, keeping in mind that the numerical model will be representative of the experimental reality. From these studies, a better understanding of the correlations between damage events that should result in nonlocal continuum modelling at the macroscale is to be expected.

## Figures and Tables

**Figure B.1:**
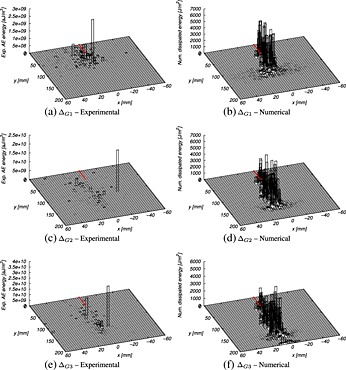
Energy maps during the different energy increments obtained from acoustic emission (AE) analysis (experimental) and mesoscale computations (numerical) for FN200 bending beam (small notch, size *D* = 200 mm). The prenotch is highlighted with a dashed line.

**Figure B.3:**
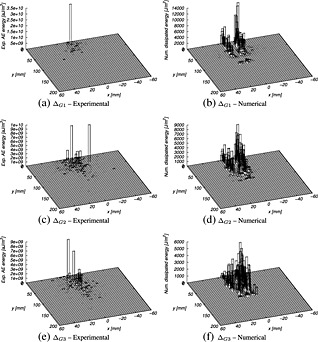
Energy maps during the different energy increments obtained from acoustic emission (AE) analysis (experimental) and mesoscale computations (numerical) for UN100 bending beam (no notch, size *D* = 100 mm).

**Figure C.1:**
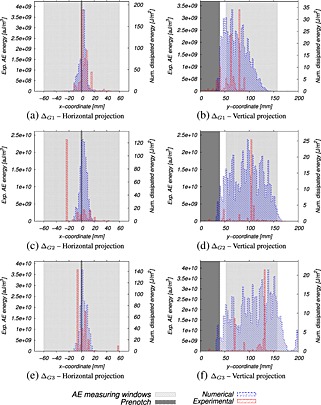
Horizontal and vertical projections of energy dissipation for FN200 bending beams (small notch, size *D* = 200 mm).

**Figure C.2:**
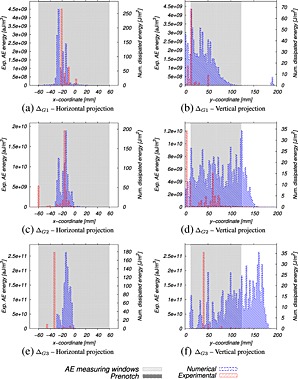
Horizontal and vertical projections of energy dissipation for UN200 bending beams (no notch, size *D* = 200 mm).

**Figure C.3:**
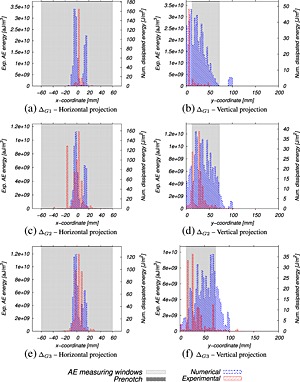
Horizontal and vertical projections of energy dissipation for UN100 bending beams (no notch, size *D* = 100 mm).

**Figure D.2:**
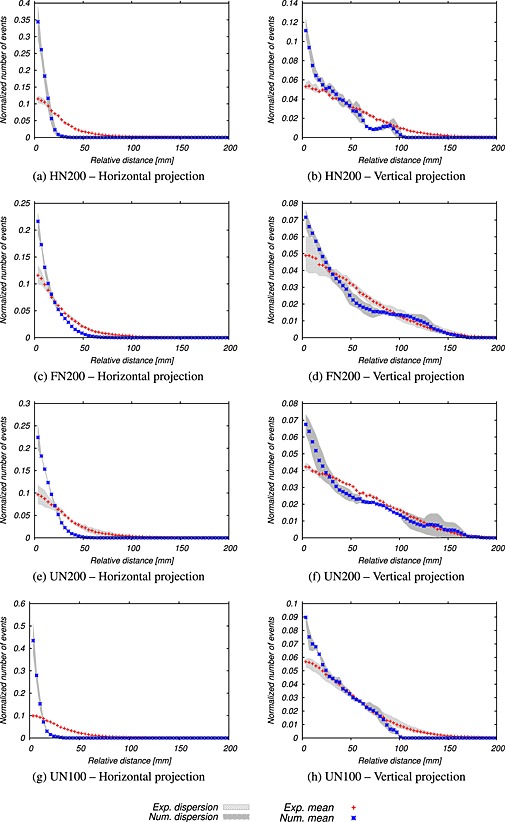
Horizontal and vertical projections of the experimental and numerical histograms in the softening phase (third energy increment Δ_*G*3_ in Figure 11).

## Footnotes

¶ The concrete mixture formulation is detailed in Table II. Experimental data and detailed gradings of the sand, the aggregates and the mix are given in 1.

## Appendix A Figures with Essential Colour Discrimination

### A.1

Certain figures in this article, particularly Figures 4, 5, 10, 13, C.1, C.2 and C.3, may be difficult to interpret in black and white. The full colour images can be found in the online version.
